# Experiences of Diagnostic Radiography Students With Workplace‐Based and Online Learning During the COVID‐19 Pandemic: A Study Across Four Higher Education Institutions in South Africa

**DOI:** 10.1002/jmrs.70031

**Published:** 2025-10-23

**Authors:** Siyabonga Goodwill Hadebe, Amina Hajat, Lynne Janette Hazell

**Affiliations:** ^1^ Department of Medical Imaging and Radiation Sciences, Faculty of Health Sciences University of Johannesburg Johannesburg South Africa

**Keywords:** diagnostic radiography students, radiography training, workplace‐based learning

## Abstract

**Introduction:**

The COVID‐19 pandemic caught the world by surprise, causing unprecedented disruptions. The training of diagnostic radiography students was particularly affected due to lockdowns and strict regulations. This study aimed to explore and describe diagnostic radiography students' workplace‐based and online learning experiences during the COVID‐19 pandemic in South Africa.

**Methods:**

Using an online open‐ended questionnaire, the data were collected from 48 fourth‐year diagnostic radiography students in 2023 from the four participating Higher Education Institutions (HEIs) in South Africa. Participants were selected through purposive sampling. Thematic analysis, supported by Atlas.Ti analysis software, was employed to analyse the data.

**Results:**

Four key themes were revealed: Theme 1: Clinical placement experiences during COVID‐19. Theme 2: Participants' personal protective equipment (PPE) experiences during COVID‐19. Theme 3: Participants' experiences of inclusive learning enablers. Theme 4: Impact of COVID‐19 lockdowns on learning experiences.

Diagnostic radiography students in South Africa faced significant challenges during clinical placements and online learning during the COVID‐19 pandemic, including fear, stress, fatigue and emotional exhaustion due to increased workloads. Online learning offered convenience but came with difficulties adapting to new methods and accessing materials, alongside technical issues.

**Conclusion:**

The findings highlight diverse experiences among diagnostic radiography students during the pandemic. This research will inform practical recommendations to improve student support during crises, helping them better navigate future similar situations.

## Graphical abstract

This study explored diagnostic radiography students' learning experiences during COVID‐19 across four South African Higher Education Institutions. Themes included challenges with clinical placements, PPE and online learning, as well as inclusive learning enablers. Findings inform strategies to support students during future crises.

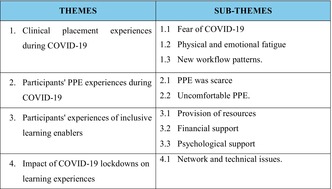

## 1 Introduction

The sudden and unexpected emergence of coronavirus disease 2019 (COVID‐19) severely strained healthcare systems worldwide and caused significant disruptions in the education sector, even in developed countries [1, 2]. Due to its highly transmissible nature, the World Health Organization (WHO) officially declared COVID‐19 a pandemic on 11 March 2020 [3]. As the COVID‐19 cases surged in South Africa, the government adopted the World Health Organization's (WHO) guidelines to combat the virus [1]. Stringent lockdown measures were implemented, leading to the suspension of classes in Higher Education Institutions (HEIs) and the closure of most establishments, except essential services such as supermarkets, pharmacies and healthcare centres [4]. Mandatory measures included face masks, social distancing, hand hygiene and a five‐level lockdown system [1, 4].

The early stages of the COVID‐19 pandemic caused significant disruptions to radiography training globally, with diagnostic radiography students experiencing interrupted clinical placements, mental health challenges and financial strain [5, 6]. Previous studies highlighted significant difficulties faced by these students, such as increased anxiety, fear of infection, inadequate personal protective equipment (PPE) and challenges in completing their syllabus [2, 5]. To maintain academic continuity amid lockdowns and restrictions on face‐to‐face teaching, HEIs worldwide, including those in South Africa, adopted online learning platforms such as Zoom Video Communications Inc., Moodle Pty Ltd., Blackboard Inc. and Microsoft Teams, Microsoft Corporation [7]. However, South African students faced additional challenges, including limited access to digital devices, high internet data costs and unstable network connectivity, which further hindered their learning experiences [7].

Despite the ongoing progression of the virus in South Africa, students in the diagnostic radiography programme, which integrates academic and workplace‐based learning (WBL), were eventually allowed to resume training at HEIs and clinical training centres [8]. In this context, WBL is an educational approach combining hands‐on experience with academic and technical skills, equipping students to face professional challenges [9]. Furthermore, the resumption of workplace‐based learning in South Africa followed a phased approach, guided by local infrastructure constraints, public health regulations and fluctuations in COVID‐19 case numbers, hospitalisations and mortality rates [4, 8].

In South Africa, HEIs offering diagnostic radiography programmes have varied clinical placement models, with some collaborating with both public and private institutions while others focus on either the public or private institutions [10]. This variation may lead to differing student outcomes, emphasising the need for research to explore these experiences. This study explored the combined experiences of diagnostic radiography students in clinical placement and online learning during the COVID‐19 pandemic, conducted across four HEIs and multiple clinical training centres. Additionally, the study provides valuable recommendations for enhancing clinical practice and supporting students during future pandemics.

## 2 Methods

This study used a qualitative, exploratory and descriptive phenomenological research design [11]. This study was conducted in four of the eight South African HEIs that provide undergraduate radiography programmes. The remaining four HEIs were excluded due to time constraints related to the dissertation submission deadline, which would have required additional time to obtain ethical and gatekeeper approvals. Three of these HEIs were not invited, while the fourth declined participation, citing students' examination commitments. The study's inclusion criteria comprised all fourth‐year diagnostic radiography students enrolled at the four participating HEIs in 2023, registered with the Health Professions Council of South Africa (HPCSA) as diagnostic radiography students. In addition, participants must have been engaged in workplace‐based learning (WBL) at an accredited HPCSA clinical training institution in the private or public sector since 2020 and be over 18 years old. In this study, ‘experiences’ are defined as the knowledge and skills gained by diagnostic radiography students through observation and hands‐on participation in WBL during the COVID‐19 pandemic, including clinical techniques, protocols, adaptations to new safety measures and online learning.

Ethical approval for this research was obtained from the University of Johannesburg HEI1 Faculty of Health Sciences Higher Degree and Research Ethics Committee (REC‐1421‐2022). Ethical permissions were also secured from the other three participating HEIs: HEI 2 (CPUT/HWS‐REC 2023/S14), HEI 3 Research Ethics Committee, and HEI 4 Research Ethics Committee (REC Ref: REC/2022/08/006).

For this study, data on the workplace‐based and online learning experiences of diagnostic radiography students during the COVID‐19 pandemic in South Africa were gathered using an online open‐ended questionnaire. The questionnaire, created with Microsoft Forms, included two sections: a brief demographic section and five open‐ended research questions designed to elicit detailed, narrative responses about the students' lived experiences. These questions were carefully developed to align with the study's qualitative aims [11], encouraging reflection on clinical learning, online learning, emotional impact and institutional support during the pandemic. Furthermore, the use of open‐ended questions within an online questionnaire format allowed participants to freely express their thoughts and perceptions, which aligns with qualitative research principles [11].

Prior to distribution to the students, a pilot study involving 18 fourth‐year diagnostic student radiographers in 2022 was conducted to assess the appropriateness, relevance and accuracy [12]. This process also aimed to refine completion time and enhance researchers' proficiency with Microsoft Forms. Data from the pilot study were excluded from the main study. The heads of departments (HODs)/research coordinators shared the questionnaire link with students, who provided electronic consent before participation. Participants had unrestricted response length and writing time, with settings allowing only one submission per person. A purposive sampling technique was used, targeting 216 fourth‐year students from these HEIs, as their clinical and online learning experiences during the COVID‐19 pandemic would provide valuable insights. A total of 52 fourth‐year students responded to the questionnaire, accounting for 24.07% of the targeted sample. Ultimately, data saturation was achieved with a final sample of 48 participants.

Detailed methods and demographic descriptions were provided to enhance transferability [13, 14]. Dependability was ensured through a comprehensive discussion guide and an open‐ended questionnaire [13]. Confirmability was achieved by employing an independent data coder to verify analysis accuracy [13, 14]. The questionnaire did not request participants' names to ensure confidentiality and anonymity.

Thematic analysis, a method for identifying patterns and themes in data, was integrated with Atlas.ti, a qualitative data analysis tool, to ensure credible research [15]. Using Braun and Clarke's six‐step approach (Table 1), this integration improved efficiency by automating tasks like text search and filtering, allowing for deeper reflection [16]. Furthermore, it enriched analytical depth by combining systematic coding with thematic analysis, ensuring robust and well‐supported interpretations [13]. The software's capability to log all actions also facilitated improved auditability, thereby enhancing the credibility of the research process [17]. Overall, this integration optimized the use of digital tools, enhancing both efficiency and depth in qualitative analysis. The process of integrating Atlas.ti with the six‐step thematic analysis approach is outlined below (Table 1).

**TABLE 1:** Integration of Atlas.ti and the six‐step thematic analysis approach of Braun and Clarke [15].

Steps	Action
Data familiarisation	The data were read and re‐read in Atlas.ti, with memos added to capture initial insights and emerging patterns.
2 Generating initial codes	Atlas.ti's coding tool was used to tag excerpts with descriptive labels (codes). Codes were applied manually to align with the research questions, while the software's search and filter features helped identify recurring phrases or concepts.
3 Searching for themes	The codes were grouped into clusters using Atlas.ti's networking and visualisation tools. Relationships between codes were explored through code co‐occurrence and conceptual mapping tools, facilitating the formation of potential themes.
4 Reviewing themes	Themes were reviewed within Atlas.ti by cross‐referencing the thematic structures with the original data. The software's query tools enabled checks for data saturation and ensured that themes were robust and comprehensive.
5 Defining and naming themes	The themes were refined and named within Atlas.ti using the memo function to document the rationale behind each theme. These memos included concise descriptions and justifications, ensuring consistency and clarity.
6 Producing the final report	Atlas.ti's export features enabled the extraction of coded data and thematic maps, which were used in the final report as visual evidence. An independent coder validated the findings by reviewing the proposal, responses and thematic report.

## 3 Findings and Discussion

### 3.1 Demographics

Demographically, there were 13 males and 35 females, with 37 participants aged 18–24 and 11 aged 25–31. Nineteen were from private clinical training institutions, and 29 were from public clinical training institutions.

### 3.2 Themes

Participants responded to the questionnaire by sharing their experiences as diagnostic radiography students, including their clinical practice, PPE supply, support from clinical training institutions, online teaching methods and learning resources. Although the open‐ended questions were designed to align with the study objectives, the coding and theme development process remained inductive. Themes were not pre‐determined by the structure of the questionnaire; however, they were allowed to emerge from the participants' responses through open coding and iterative thematic development. In line with qualitative research standards, an independent coder reviewed the coding and thematic structure to ensure the accuracy and trustworthiness of the final themes. The following four themes emerged: Theme 1: Clinical placement experiences during COVID‐19. Theme 2: Participants' PPE experiences during COVID‐19. Theme 3: Participants' experiences of inclusive learning enablers. Finally, Theme 4: Impact of COVID‐19 lockdowns on learning experiences. The following table (Table 2) presents the themes and categories that emerged during data analysis.

**TABLE 2:** Themes and sub‐themes.

Themes	Sub‐themes
Clinical placement experiences during COVID‐19	1.1 Fear of COVID‐19 1.2 Physical and emotional fatigue 1.3 New workflow patterns
2 Participants' PPE experiences during COVID‐19	2.1 PPE was scarce 2.2 Uncomfortable PPE
3 Participants' experiences of inclusive learning enablers	3.1 Provision of resources 3.2 Financial support 3.3 Psychological support
4 Impact of COVID‐19 lockdowns on learning experiences	4.1 Network and technical issues

#### 3.2.1 Theme 1: Clinical Placement Experiences During COVID‐19

Diagnostic radiography students in South Africa experienced various challenges during their clinical placements amidst the COVID‐19 pandemic. The pandemic was brutal for everyone, even without direct contact with patients. The training of diagnostic radiography students involves hands‐on work with patients [18], which increases their vulnerability to virus exposure and heightened emotional strain [19]. These emotions became even stronger, with COVID‐19 being highly contagious and dangerous. When the diagnostic radiography students were asked, ‘Describe your experience as a diagnostic radiography student during the COVID‐19 pandemic.’ The participants reported heightened stress and anxiety during the COVID‐19 pandemic, primarily due to the fear of contracting the virus.

Stepping into the hospital environment after months of lockdown brought me anxiety, especially after having read on the news all the information about the virus. Participant 19, HEI 1, Public

Very stressful most of the time. Coming to work gave me stress that I will catch Coronavirus. Participant 47, HEI 4, Private

It was very stressful for me as a student. I had to work while thinking about the virus in the back of my mind. It was not nice at all working at that time. Participant 48, HEI 3, Private

##### 3.2.1.1 Sub‐Theme 1.1: Fear of COVID‐19

Many participants described their experiences during the COVID‐19 pandemic as dominated by fear. This fear stemmed from uncertainty, health risks and the challenges of adapting to new environments. The anxiety of continuing education and clinical practice under unprecedented conditions and concerns for their patients' well‐being made fear a central part of their experience [18]. Participants also feared contracting and spreading the virus to vulnerable family members, which added to their distress. Furthermore, the fear was intensified by the unpredictability of the pandemic and limited knowledge about the long‐term effects of the virus [6]. This persistent fear affected their confidence in engaging with patients and carrying out their clinical duties, making their training experience more daunting. For example, participants affirmed;

It was very tough as you didn't know what to expect and what you were meant to do with the patients, scary moments. Participant 7, HEI 1, Private

It was the scariest thing I have ever experienced. Not knowing what the outcome of the patients you X‐rayed was scary. Participant 8, HEI 1, Public

It was incredibly difficult in the beginning since everyone was concerned about their lives and loved ones. I was scared of contracting the virus or taking it home to family members. Participant 9, HEI 1, Public

It was hectic. Sometimes, we were not allowed to go to areas like resus unit or casualty. Gaining experience was difficult because we were working in fear. Participant 18, HEI 1, Public

##### 3.2.1.2 Sub‐Theme 1.2: Physical and Emotional Fatigue

The COVID‐19 pandemic posed significant physical dangers to the participants, who faced unprecedented challenges during their clinical training. As they continued their training, they were frequently exposed to the virus, significantly increasing their infection risk. This turned their training from a standard learning experience into a time of significant health risk and danger, as emotional and physical fatigue took its toll [18]. The participants reported feeling physically and emotionally fatigued, as they were expected to meet high demands during their clinical placements [2, 18].

Insane, when COVID‐19 started, I was a repeating 1st year for applied physics. My private practice made me work even more since we went online. I learned a lot from my clinical training, but it was very draining on my physical and emotional side. Participant 1, HEI 1, Private

Draining, exhausting each day, emotional, and sometimes painful. Participant 42, HEI 4, Private

The study revealed that some participants were expected to have advanced knowledge despite missing clinical time. The pressure to catch up on missed practical experience while meeting academic expectations added to their fatigue, making the learning process more challenging.

It was really bad. I started my clinical practice late, around September 2020, instead of July, and was expected to know more of the content. It was really draining. We were expected to know chest X‐ray on that very September and get assessed on it. Participant 4, HEI 1, Public

I'd describe my experience as being difficult. We only started our clinical training in the second year and worked many more hours to make up for the lost time. It felt like we were thrown into the deep end and required to work regardless because we were healthcare students. Participant 20, HEI 3, Private

I had a difficult clinical experience. In our first year, we did not attend clinicals due to COVID‐19, as cases were still high. When we finally started in our second year, we were only allowed one week of clinical training per month. This was not enough, as we faced immense pressure to cover both first‐year (normal views) and second‐year (specialised views) content within a limited time. Participant 34, HEI 2, Public

##### 3.2.1.3 Sub‐Theme 1.3: New Workflow Patterns

During the COVID‐19 pandemic, diagnostic radiography students faced significant challenges adapting to updated workflows and protocols. As new measures were introduced, students had to quickly adjust their routines and learn new systems, often leading to confusion and difficulty in implementation [2]. Frequent updates to infection control measures, increased PPE use and social distancing requirements created uncertainty and disrupted established routines, adding to their stress [2, 5, 8]. Balancing these changes with concerns for their own health and safety made the transition even more challenging, impacting their ability to integrate the new processes into their daily clinical practice [8]. The pressure to maintain efficiency while ensuring safety contributed to heightened anxiety, affecting their confidence and training experience.

It was scary that we had to adapt to new methods of interacting with patients. Participant 2, HEI 1, Private

It was challenging at first to change how we work and learn how to keep safe. Participant 15, HEI 1, Private

As a 1st‐year student in private practice, I was still expected to attend work. I experienced chaos with new protocols and policies on COVID‐19. I felt overwhelmed and scared. Participant 38, HEI 2, Private

#### 3.2.2 Theme 2: Participants' PPE Experiences During COVID‐19

Following the disruption of clinical training, radiography students in South Africa were required to resume their practical experience in a safe environment while ensuring quality patient care. However, like many healthcare workers, they faced significant PPE‐related challenges [19, 20]. Inadequate PPE training left many students unprepared for crucial tasks such as donning and doffing PPE, infection control and managing COVID‐19 patients. A study by Haegdorens et al. [21] suggests that comprehensive PPE training can help reduce anxiety and emotional distress among healthcare workers. Similarly, the World Health Organization stresses the importance of proper training for all healthcare workers, including students, to improve preparedness and safety [20]. The lack of consistent protective measures and sufficient support made clinical training during the pandemic overwhelming and difficult for students to navigate.

I was not familiar with the PPE at first because we didn't get any session about it in my practice. Participant 4, HEI 1, Public

There was never a training on how to deal with covid patients. As a student I was informed that when a covid resus portable comes I must just go do it, I never received proper training so what I knew was what I leant on the news. Participant 8, HEI 1, Public

We were not taught how to wear the PPE and how long the PPE should be worn. Participant 18, HEI 1, Public

There was not much training we had to try and figure out everything on our own because everyone was under pressure. Participant 20, HEI 3, Private

##### 3.2.2.1 Sub‐Theme 2.1:PPE Was Scarce

The persistent shortages of PPE continued to be a significant source of concern for the participants. These conditions not only increased their emotional distress but also created a sense of inadequacy as they struggled to perform their duties under these challenging and unsafe circumstances [19, 22]. The World Health Organization [22] has also highlighted that PPE shortages worldwide have contributed to healthcare workers' heightened vulnerability and stress, further amplifying the challenges in training environments like these. This issue was exacerbated by the requirement to reuse protective equipment, which increased their anxiety about maintaining proper hygiene and safety standards [22]. Furthermore, the restriction that only qualified radiographers and fourth‐year students were allowed to wear PPE made the participants feel even more vulnerable and exposed, as they were left without adequate protection during their clinical placements.

There was a shortage of PPE at some point; however, when it was available, it did not last long. Participant 5, HEI 1, Private

In public hospitals, there were often shortages of PPE. So, we would have to reuse the gowns. Which was ultimately hazardous to me as a student Radiographer. Participant 11, HEI 1, Public

The only people allowed to wear PPE were qualified radiographers and fourth‐year students. Participant 12, HEI 1, Private

The PPE shortages affected the majority of the participants, raising concerns about increased COVID‐19 risk and severe illness. The global shortage resulted from high demand and supply gaps, leading the WHO to urge prioritisation of PPE distribution for students [20, 22]. During PPE shortages, allegations surfaced of funds for PPE and medical equipment being misappropriated by South African government officials and private companies [23, 24]. A study by Hazell and Stork [19] emphasised the need for stricter measures to prevent future shortages, stressing the importance of oversight and accountability.

##### 3.2.2.2 Sub‐Theme 2.2: Uncomfortable PPE

During the COVID‐19 pandemic, participants experienced significant discomfort while wearing PPE during clinical training. Davey et al. [25] indicate that while PPE is essential for protection, wearing it for extended periods imposes physical strain, making it difficult to maintain focus and perform duties efficiently. Furthermore, earlier studies have indicated that prolonged use of PPE caused excessive sweating and dehydration, negatively affecting well‐being [25]. Participants reported that PPE felt uncomfortably hot and intimidating. Facemasks, in particular, made breathing difficult, leading to fatigue and reduced concentration during clinical tasks.

The PPE was very hot and uncomfortable. Participant 3, HEI 1, Public

Very suffocating, but I had to think of my life that comes once. Participant 22, HEI 2, Public

PPE is the hottest, most uncomfortable and scariest thing I've ever worn. You feel scared and look the part to other people when you're in PPE. It takes forever to get into it, but you still don't feel safe enough wearing it somehow. Participant 30, HEI 3, Public

Having to change PPE with each patient I encountered was not great, and the fact that it was hot was not helping at all. Participant 41, HEI 4, Private

A South African study by Khan et al. [26] found that PPE not only caused physical discomfort but also hindered communication. Davey et al. [25] further highlight that the restrictive design of PPE limited movement, making certain procedures more challenging and time‐consuming. Additionally, prolonged PPE use contributed to psychological strain, increasing stress and anxiety as participants contended with both physical discomfort and the emotional demands of their clinical roles [24]. These challenges exacerbated an already demanding training environment, requiring participants to balance essential protection with the difficulties associated with PPE use.

#### 3.2.3 Theme 3: Participants' Experiences of Inclusive Learning Enablers

During the lockdown restrictions, online learning provided a significant advantage regarding convenience and flexibility. With traditional face‐to‐face classes suspended, students and educators could continue their educational activities from home [18]. This mode of learning allowed participants to manage their time better, access resources remotely, and learn at their own pace and in the comfort of their own space. Moreover, the prerecorded lectures, additional study materials, and the ability to rewind videos allowed students to understand the content better.

The online teaching methods were helpful as we had PowerPoints with voice overs in them to allow us to go back and revise for tests and the online classes were recorded for us. Participant 14, HEI 1, Public

Online learning was the absolute best. Learning at our comfort zone and at our own pace helped me perform even better. Participant 16, HEI 1, Public

It had it benefit like recording the lecture and rewatch it later. Participant 21, HEI 3, Public

Our lecturers ensured to provide us with all the learning materials we needed which made our learning easier. Participant 23, HEI 3, Public

##### 3.2.3.1 Sub‐Theme 3.1: Provision of Resources

The provision of resources by the government and HEIs was crucial for enabling students to continue their learning amid the COVID‐19 pandemic. These resources included access to online learning platforms, educational materials and support services, which helped students adapt to online learning and maintain their academic progress [27]. The government and HEIs played a crucial role in supporting students, helping them overcome the challenges of the pandemic and continue their studies effectively despite the disruptions to traditional learning environments [27]. The students mentioned that they were provided with free internet data bundles and laptops.

We got data from the university, and students needing laptops got them to help them study. Participant 14, HEI 1, Public

I received a laptop from the university and also internet data during the period of online learning which immensely assisted with this method of learning. Participant 17, HEI 1, Public

Those with no online learning devices were given devices such as laptops by the university. The data was also given to us to access online learning. Participant 18, HEI 1, Public

Additionally, participants mentioned that they were provided access to Virtual Private Networks (VPNs), enabling them to connect to private networks securely while studying from home. This ensured their online activities remained safe, safeguarded sensitive information, and provided smooth access to educational resources and communication with faculty and peers [28]. The use of VPNs helped students continue their studies without concerns about digital security during the pandemic.

They also introduced the use of VPN, which allowed us to access all the student portals for free, e.g., student email, SOS, and Blackboard. Participant 37, HEI 2, Public

The university gave us access to eduroam hotspots and zero‐rated student VPNs. Participant 38, HEI 2, Private

##### 3.2.3.2 Sub‐Theme 3.2: Financial Support

Financial support enabled students to continue their education during the COVID‐19 pandemic. The National Student Financial Aid Scheme (NSFAS), a South African government‐funded programme providing financial assistance to underprivileged students, ensured the continuation of allowances for tuition, living and accommodation expenses [29, 30]. This support was critical in helping students manage increased financial pressures, such as higher technology costs for online learning and the need for additional resources to adapt to remote education [31]. By alleviating financial insecurity, these allowances allowed students to focus on their studies despite the challenges posed by the pandemic.

We still got our stipend, which helped a lot during a time of need. Participant 5, HEI 1, Private

NSFAS continued paying us, which was helpful as it allowed me to buy data when I ran out of it. Participant 21, HEI 3, Public

They ensured we had a stipend for going to work. Participant 39, HEI 4, Private

##### 3.2.3.3 Sub‐Theme 3.3: Psychological Support

During the COVID‐19 pandemic, some diagnostic radiography students received official psychological support to help them cope with the added stress and challenges of their training. This support included counselling services, mental health resources and programmes designed to address the emotional and psychological impact of the pandemic [6]. The participants who received this support noted that it fostered a sense of unity, as they could support one another and share their experiences with their seniors. This support was crucial for maintaining emotional well‐being during such a demanding time [6, 32]. Participants appreciated the availability of these services, as they helped them manage the heightened stress and uncertainty brought about by the pandemic.

Counselling was offered to talk about our experiences with the pandemic. We remained a tight‐knit family and grew even closer trying to support each other. Participant 5, HEI 1, Private

We had an open‐door policy on sharing our challenges with our superiors. Participant 15, HEI 1, Private

Occupational stress course provided by my clinical practice. I was often reminded that online student counselling services were available. As well as webinars and workshops on grief, mental health, coping, and suicide awareness. Participant 38, HEI 2, Private

The study revealed that only participants from private clinical training institutions had access to psychological support, unlike those from public institutions, which placed them in a disadvantaged position. Research indicates that such support enhances well‐being and coping skills during challenging times [33]. When management provides psychological support, it improves relationships and workplace morale and reduces absenteeism [34].

#### 3.2.4 Theme 4: Impact of COVID‐19 Lockdowns on Learning Experiences

During the COVID‐19 pandemic, lockdown restrictions created significant learning barriers for some participants, making it challenging to access educational resources and maintain their studies. The closure of HEIs and the shift to online learning posed difficulties in adapting to new modes of instruction and accessing necessary materials [7, 30]. These barriers disrupted their educational progress and added to the stress and frustration of navigating a rapidly changing academic landscape [7, 31]. As a result, some participants struggled to keep up with their coursework amidst the pandemic's many challenges.

##### 3.2.4.1 Sub‐Theme 4.1: Network and Technical Issues

During the COVID‐19 pandemic, students in South Africa faced major network and technical issues during online learning [7]. The technical issues and lack of network connectivity led to difficulties for some participants in accessing learning materials promptly or obtaining clear audio and video content. These challenges, compounded by socio‐economic factors and geographic location, hindered effective participation in online classes [18]. Despite efforts by the government, the South African Department of Higher Education and Training (DHET), and HEIs to support online learning, these technical difficulties added stress and made it harder for students to keep up with their coursework.

It was very challenging, especially because most of our classes were online, and we were at home most of the time, meaning we struggled with connectivity. Participant 45, HEI 4, Private

Well after the covid hit we switch to online learning completely. At that, I was in my first year, and I was not familiar with online learning and Zoom meetings for group work. The network from rural areas was really bad. Sometimes, I will miss some classes because of the network. Participant 18, HEI 1, Public

Kinda difficult to understand at first. And the network was also a big problem. The network will come and go or become slow. Participant 47, HEI 4, Private

Furthermore, frequent load‐shedding (electrical power interruptions occur in South Africa on a scheduled daily basis) increased participants' frustration. The power utility implemented these electrical power interruptions to alleviate strain on underperforming power grids, leaving the participants without electricity for extended periods [35].

It was very difficult; the network was an issue. I experienced difficulties attending online classes because of network problems and load shedding (electrical power interruption). Participant 25, HEI 1, Private

We had so many issues, and load‐shedding (electrical power interruption) gave me headaches. Participant 34, HEI 4, Private

The lack of infrastructure investment highlighted a rural–urban divide in online learning [36]. Bridging this gap will require significant government and private sector investment to prevent future challenges [7]. Despite these difficulties, students appreciated the support of the government and HEIs, such as providing laptops and data bundles during the transition to online learning.

The study suggests that diagnostic radiography students in South Africa had varied experiences during the COVID‐19 pandemic. Many participants reported feelings of fear, stress and fatigue due to the sudden threat posed by the virus, which extended to concerns for their loved ones. They faced demanding conditions that required constant adaptation to unexpected changes and increased workloads, leading to physical and emotional exhaustion. Similar findings were observed in studies conducted in Singapore and Europe during the pandemic [2, 5]. However, these studies highlighted a lack of financial support, which was not an issue in this study. Visser and Law‐van Wyk [6] propose that to overcome these challenges, management in both public and private clinical training institutions should enhance work schedules, facilitate regular breaks, address staff shortages, strengthen organisational support and ensure access to mental health services for all students, including those in public institutions.

## 4 Limitations

This study was conducted in four out of eight HEIs offering radiography programmes in South Africa based in Gauteng, KwaZulu‐Natal and the Western Cape. Time constraints limited a more detailed description by including all eight HEIs. Furthermore, accessibility challenges, including the study's overlap with exam periods and logistical issues, necessitated the use of a written format instead of face‐to‐face participation. Follow‐up studies post‐qualification could assess long‐term effects. Future research should encompass all radiography disciplines and HEIs for a comprehensive conclusion.

## 5 Recommendations

The following recommendations are suggested to enhance support for students during a pandemic.

- A 24‐h psychological support system should be readily accessible to students, possibly online.
- Clinical tutors in clinical placement should foster an open‐door policy to encourage dialogue and address student concerns.
- Clear and consistent communication with students on upcoming workflow changes is essential, and students should have opportunities to provide feedback.
- Investing in local PPE manufacturing would enable quick, reliable supply access during a pandemic.
- Regular daily stock assessments, alongside timely ordering and delivery would ensure constant availability.
- Additionally, collaboration between the government and the private sector to develop rural infrastructure can significantly reduce barriers to students accessing online learning.
- Network providers should prioritise improving rural internet services and establish dedicated rural networks to bridge the digital divide.

## 6 Conclusion

This study explored diagnostic radiography students' experiences with workplace‐based and online learning during the COVID‐19 pandemic in South Africa. Students faced numerous challenges, including rapidly adapting to new safety protocols, dealing with PPE shortages and managing the constant fear of infection. The stress of clinical placements was compounded by the uncertainty of the pandemic, emphasising the importance of mental health support. The provision of laptops underscored the need for greater government support and better rural infrastructure to enhance learning. Despite these obstacles, students demonstrated resilience, learning to manage increased workloads and emotional strain. They also benefited from the government's and HEIs' efforts to facilitate the transition to online learning, appreciating its convenience and flexibility during this challenging period. This experience underscored the importance of adaptability and support systems in balancing training with the pressures of the pandemic.

## Conflicts of Interest

The authors declare no conflicts of interest.

## Data Availability

Data openly available in a public repository that issues datasets with DOIs.
